# Associations between Cloninger’s temperament and character traits and light preference

**DOI:** 10.3389/fpsyt.2025.1605581

**Published:** 2025-09-22

**Authors:** Hirofumi Hirakawa, Takeshi Terao, Kentaro Kohno, Akari Sakai, Nobuko Kawano

**Affiliations:** ^1^ Department of Neuropsychiatry, Faculty of Medicine, Oita University, Yufu, Oita, Japan; ^2^ Oita Occupational Health Management Center, Nishinihon Occupational Health Center, Oita, Oita, Japan; ^3^ Department of Psychology, Faculty of Welfare and Health Science, Oita University, Oita, Oita, Japan

**Keywords:** light preference, brightness, darkness, temperament, harm avoidance, self-directedness

## Abstract

**Background:**

It is unknown whether light preference (brightness or darkness) is associated with Cloninger’s temperament and character traits. This study examined the association between Cloninger’s temperament, character, and light preference in healthy individuals. The aim of the present study was to investigate the hypotheses that self-transcendence but not self-directedness may be associated with brightness preference, whereas harm avoidance may be associated with darkness preference.

**Methods:**

Data from 130 healthy participants were analyzed in an opt-out study. First, the data distribution of temperament and character scores was investigated by Shapiro-Wilk test. If the distribution was not normal, we used non-parametric test to compare temperament and character scores based on light preference (brightness or darkness), morning light exposure (yes or no), and bedtime mobile phone use (yes or no). Second, binomial logistic regression analyses were performed for each temperament and character scores as dependent variables in which the subjects were divided into two groups using median as a cut-off point (less than median = 0, equal to or more than median = 1), with age, sex, light preference, morning light exposure, and bedtime mobile phone use as independent variables.

**Results:**

Self-directedness was significantly associated with brightness preference and no bedtime mobile phone use. Our hypotheses were not supported.

**Conclusions:**

The present findings suggest that self-directedness may be associated with brightness preference and no bedtime mobile phone use. Further studies are required to determine the causal relationships.

## 1 Introduction

Since Cloninger et al. (1) described a psychobiological model of temperament and character with four temperament dimensions (novelty seeking, harm avoidance, reward dependence, and persistence) and three character dimensions (self-directedness, cooperativeness, and self-transcendence), many studies have utilized this model with the Temperament and Character Inventory (TCI). Regarding the association between Cloninger’s temperament and character model with depression and suicide, Lee et al. (2) suggested that self-directedness may mitigate the effect of depressive mood on suicide risk. Meanwhile, Takanobu et al. (3) suggested that high harm avoidance, low self-directedness, and low cooperativeness may predict depression and suicidal ideation. In terms of treatment outcomes, Balestri et al. (4) reported that non-remitters of depression tend to have high harm avoidance and self-transcendence, along with low persistence and self-directedness, whereas non-responders of depression exhibit high harm avoidance and low reward dependence and self-directedness. Tsigkaropoulou et al. (5) suggested that high resilience is associated with low harm avoidance and high persistence in depression, low harm avoidance in bipolar disorder, and high persistence and self-directedness in healthy individuals.

As for recent studies regarding TCI, Komasi et al. (6) performed a meta-analysis of 149 studies and showed that high harm avoidance and low self-directedness played a fundamental role in psychopathology. They suggest that harm avoidance is a biological trait associated with the behavioral inhibition system, and seems to have a strong relationship with psychopathology. Although harm avoidance has been proposed as an independent trait of other temperament and character traits, it may have a negative effect on self-directedness. Conversely, low self-directedness may facilitate psychopathology if combined with high levels of harm avoidance. Lee et al. (7) investigated 287 Korean university students and showed that novelty-seeking and persistence temperaments have demonstrated the moderating effect in the association between morningness and well-being, and that harm avoidance temperament and self-directedness character sequentially mediated the relationship between morningness and well-being. Oh and Cloninger (8) investigated 1384 Korean adults over 18 years old (58% female) and showed that in anxiety disorder, harm avoidance and reward dependence were higher than in depressive disorder, and self-directedness was higher than in anxiety disorder + depressive disorder; in depressive disorder, persistence, self-directedness and cooperativeness were higher than in anxiety disorder + depressive disorder; and in anxiety disorder + depressive disorder, harm avoidance was highest and persistence and self-directedness were lowest (i.e., they were lowest in Resilience). Lavonius et al. (9) investigated whether polygenic risk scores (PRS) for depression predicts trajectories of temperament traits from early adulthood to middle age in participants who came from the population-based Young Finns Study (n =2212). As a result, High PRS significantly predicted higher persistence from early adulthood to middle age when controlling for depressive symptoms, psychosocial family environment, and socioeconomic factors. PRS did not predict trajectories of novelty seeking or reward dependence. Additionally, they found an interaction between polygenic risk scores for depression and depressive symptoms when predicting the harm avoidance subscale anticipatory worry, indicating that the association of anticipatory worry with depressive symptoms is stronger in individuals with higher (vs. lower) PRS for depression. Erdem and Bahadir (10) investigated 102 adolescents with attention deficit hyperactivity disorder (ADHD) aged 11–17 and 101 age and gender matched healthy controls, and showed that ADHD participants scored higher in novelty seeking and harm avoidance, while controls exhibited greater persistence and self-directedness. Additionally, novelty seeking was pronounced in those with an evening chronotype and positively correlated with sleep disturbances, including difficulties initiating and maintaining sleep.

On the other hand, as for the association between light and mood disorder, bright light therapy in the morning or exposure to ambient daylight may improve depression, whereas dark or virtual darkness therapy may improve mania (11, 12). In addition, greater daytime light exposure in daily life may be associated with less depressive symptoms in bipolar disorder (13), whereas bedroom light exposure at night may be significantly associated with manic symptoms in bipolar disorder (14). In addition, illuminance may affect risk preferences, ambiguity preferences, choice consistency and dominance violations (15). Moreover, suicide rates may be negatively correlated with yearly sunshine, with an increased risk observed in regions with fewer daylight hours (16). It is suggested that the suprachiasmatic nucleus (SCN) independent pathway, which links intrinsically photosensitive retinal ganglion cells (ipRGCs) to the perihabenular nucleus, regulates mood through light exposure, whereas ipRGCs projecting to the SCN mediate the effects of light on circadian rhythms and learning (17). Also, four weeks of bright light exposure increased the uptake of ^18^F-fluorodeoxyglucose in the left hippocampus and the left hippocampal dentate gyrus head volume in healthy participants (18, 19) and in patients with mood disorders (20). Therefore, the left hippocampus may be also involved in the effects of light.

Considering the above evidence, light may play an important role in the pathophysiology of mood disorders. Additionally, with regard to the association between light and temperament, hyperthymic temperament has been positively associated with daytime exposure (21) and higher illuminance may maintain hyperthymic temperament via light effects on brain function in a dose-dependent manner (22), whereas cyclothymic temperament has been negatively associated with daytime illuminance (23). In addition, more hyperthymic subjects may prefer brightness and un-prefer darkness than less hyperthymic subjects (i.e., heliotropism) (24). Therefore, it seems likely that light may maintain hyperthymic temperament and conversely individuals with hyperthymic temperament may prefer light, suggesting bidirectional relationship between hyperthymic temperament and light.

Our recent research suggests that depressive, cyclothymic, and anxious temperaments may be associated with a preference for darkness, whereas hyperthymic temperament may be associated with a preference for brightness (25). As for the associations between affective temperaments and Cloninger’s temperament and character, we showed that hyperthymic temperament scores were significantly and positively associated with self-transcendence scores, but not with self-directedness scores after adjustment for relevant factors, and that harm avoidance scores were significantly and negatively associated with hyperthymic temperament scores and self-directedness scores, but not with self-transcendence scores after adjustment for relevant factors (26). Also, we showed that harm avoidance scores were significantly and positively associated with anxious temperament scores after adjustment for relevant factors (27). Therefore, it can be hypothesized that self-transcendence but not self-directedness may be associated with brightness preference, whereas harm avoidance may be associated with darkness preference. The aim of the present study was to investigate these hypotheses.

## 2 Subjects and methods

### 2.1 Subjects

We analyzed data from a previous study (28) on psychotherapy in apparently healthy participants. The inclusion criterion was individuals aged 20 years or older who provided written informed consent. Participants were recruited via electronic and physical bulletin boards as well as flyers. Individuals with serious psychiatric disorders, as determined by the Mini-International Neuropsychiatric Interview (M.I.N.I.), were excluded from the study. All participants in this study were considered healthy. This opt-out study included data from 130 healthy participants, which included 108 females and 22 males, with a mean age of 49.3 years (standard deviation [SD]: 12.1). The study was approved by the Ethics Committee of Oita University Faculty of Medicine on June 12, 2023 (approval number 2536).

### 2.2 Cloninger’s temperament assessment

The participants completed the Japanese version of the TCI, which assessed temperament using four subscales—novelty seeking, harm avoidance, reward dependence, and persistence—and character using three subscales—self-directedness, cooperativeness, and self-transcendence (1). The reliability and validity of the Japanese version of the TCI have been previously established (29, 30), which showed Cronbach *α*: 0.69-0.85 for the temperament scales; 0.81-0.82 for the character scales, and test-retest correlations (1- to 2-mo interval): 0.72-0.84 for the temperament scales; 0.72-0.78 for the character scales.

### 2.3 Light preference and behaviors

As a whole, the questionnaire consisted of 12 domains of life habits (physical activity, music listening, reading habit, meditation, religion, smoking, diet, alcohol drinking, sleep, work, light, unforgettable hardship). So far, the data of the questionnaire on life habits was used in two opt-out studies (25, 31). The domain of light in the questionnaire included three questions assessing light preference (brightness or darkness), morning light exposure (yes or no), and bedtime mobile phone use (blue light exposure) (yes or no). In the concrete, there was the following questions: “We ask you about the relationship with light as follows. 1) Which do you prefer, brightness or darkness? (brightness or darkness); 2) Do you have the habit in receiving morning light? (Yes or No); 3) Do you have the habit in using mobile phone at bedtime? (Yes or No)”. For these answers, we replaced brightness for 1 and darkness for 2, and Yes for 1, and No for 0 in order to use the answers for statistical analyses. It has been shown that depressive, cyclothymic, and anxious temperaments may be associated with a preference for darkness, whereas hyperthymic temperament may be associated with a preference for brightness (25). The present study intended to extend the study to TCI with the new hypothesis that self-transcendence but not self-directedness may be associated with brightness preference, whereas harm avoidance may be associated with darkness preference.

### 2.4 Data analysis

First, the data distribution of temperament and character scores was investigated by Shapiro-Wilk test. If the distribution was not normal, we used non-parametric test to compare temperament and character scores based on light preference (brightness or darkness), morning light exposure (yes or no), and bedtime mobile phone use (yes or no). Second, binomial logistic regression analyses were performed for each temperament and character scores as dependent variables in which the subjects were divided into two groups using median as a cut-off point (less than median = 0, equal to or more than median = 1), with age, sex, light preference, morning light exposure, and bedtime mobile phone use as independent variables.

## 3 Results

**Table 1** showed the data distribution of Cloninger’s Temperament and Character of our participants, where the distribution was not normal in harm avoidance, reward dependence, persistence, self-directedness, cooperativeness, or self-transcendence, other than novelty seeking scores.

**Table 1:** Distributions of Cloninger’s temperament and character.

TCI	N	MIn	Max	Mean	SD	Median	Shapiro-Wilk test p
Novelty Seeking	128	12	32	21.3	4.7	21	0.135
Harm Avoidance	125	4	32	18.8	6.8	19	0.007
Reward Dependence	129	2	22	15.1	3.7	15	0.020
Persistence	128	0	8	4.5	2.1	4	<0.001
Self-directedness	125	8	41	28.8	7.8	30	<0.001
Cooperativeness	126	9	38	28.1	5.2	29	<0.001
Self-transcendence	129	3	27	12.5	6.1	12	<0.001

As shown in **Table 2**, participants with darkness preference scored significantly higher harm avoidance scores than those with brightness preference. Participants who reported morning light exposure had significantly higher self-directedness scores than those without morning light exposure. Bedtime mobile phone users had significantly lower self-directedness and self-transcendence scores than non-users.

**Table 2:** Associations between light preference, behavioral factors, and Cloninger’s temperament and character.

TCI	Light Preference (Brightness vs. Darkness)	Effect size	95% confidence interval	Non-parametric test (p)
Novelty seeking	21.4 (4.8) vs. 20.4 (4.5)	0.22	-0.22 ∼0.67	0.93
Harm avoidance	17.8 (6.5) vs. 23.1 (6.6)	-0.81	-1.28 ∼-0.34	0.022
Reward dependence	15.3 (3.6) vs. 14.0 (3.9)	0.34	-0.10 ∼0.79	0.74
Persistence	4.5 (2.1) vs. 4.0 (2.0)	0.25	-0.21 ∼0.70	0.59
Self-directedness	29.5 (7.6) vs. 25.9 (8.3)	0.47	0.01 ∼0.93	0.078
Cooperativeness	28.1 (5.5) vs. 28.5 (3.5)	-0.08	-0.54 ∼0.37	0.56
Self-transcendence	12.8 (6.2) vs. 11.8 (5.6)	0.17	-0.27 ∼0.62	0.71
	Morning Light Exposure (Yes vs. No)	Effect size	95% confidence interval	Non-parametric test (p)
Novelty seeking	21.6 (4.7) vs. 20.3 (5.0)	-0.27	-0.67 ∼0.13	0.81
Harm avoidance	18.2 (6.7) vs. 20.5 (6.8)	0.35	-0.06 ∼0.75	0.22
Reward dependence	15.4 (3.7) vs. 14.2 (3.5)	-0.34	-0.73 ∼0.06	0.28
Persistence	4.7 (2.1) vs. 3.7 (1.9)	-0.47	-0.87 ∼-0.07	0.12
Self-directedness	29.5 (8.0) vs. 27.1 (6.9)	-0.31	-0.72 ∼0.10	0.049
Cooperativeness	28.4 (5.3) vs, 27.4 (4.8)	-0.20	-0.59 ∼0.20	0.29
Self-transcendence	13.3 (6.2) vs. 10.1 (4.9)	-0.54	-0.93 ∼-0.14	0.32
	Bedtime Mobile Phone Use (Yes vs. No)	Effect size	95% confidence interval	Non-parametric test (p)
Novelty seeking	22.7 (5.1) vs. 20.3 (4.3)	-0.51	-0.87 ∼-0.15	0.14
Harm avoidance	20.3 (6.9) vs. 17.8 (6.6)	-0.38	-0.74 ∼-0.02	0.078
Reward dependence	15.3 (3.7) vs. 14.9 (3.7)	-0.09	-0.44 ∼0.26	0.54
Persistence	4.1 (2.2) vs. 4.7 (2.0)	0.29	-0.07 ∼0.64	0.43
Self-directedness	27.6 (7.1) vs. 29.9 (8.2)	0.30	-0.06 ∼0.66	0.027
Cooperativeness	28.0 (5.1) vs. 28.2 (5.3)	0.03	-0.33 ∼0.38	0.80
Self-transcendence	10.9 (5.8) vs. 13.5 (6.0)	0.43	0.07 ∼0.78	0.019

Values are presented as mean (standard deviation).

**Figure 1:**
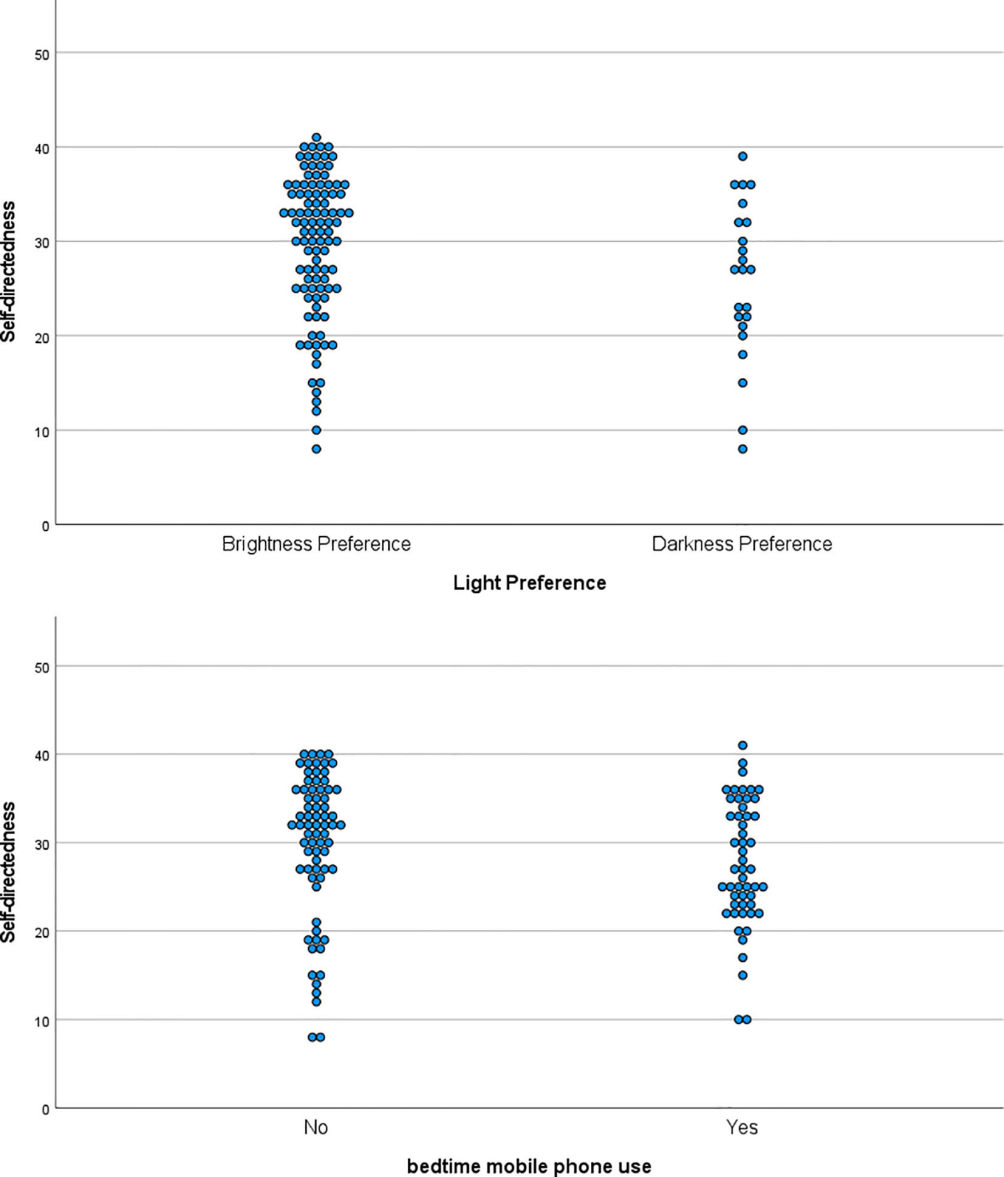
Self-directedness, brightness preference, and no bedtime mobile phone use. Self-directedness was significantly associated with brightness preference than darkness preference. Self-directedness was significantly associated with no bedtime mobile phone use.

As shown in **Table 3**, the significance of the model was not significant in novelty seeking, harm avoidance, reward dependence, or self-transcendence, but significant in persistence, self-directedness, and cooperativeness. However, there were no significant factors such as light preference, morning light exposure, and bedtime mobile phone use in persistence or cooperativeness. Only in self-directedness, darkness preference and bedtime mobile phone were significantly and negatively associated with self-directedness. **Figure 1** shows the associations of self-directedness with brightness preference and no bedtime mobile phone use.

**Table 3:** Binomial logistic regression analyses of light preference and behavioral factors in Cloninger’s temperament and character.

Novelty seeking	B	Exp (B) (95% CI)	p
Gender (Female = 1, Male = 2)	0.34	1.40 (0.50 ∼3.98)	0.53
Age	0.035	1.04 (1.00 ∼1.07)	0.042
Light preference (Brightness = 1, Darkness = 2)	0.098	1.10 (0.42 ∼2.87)	0.85
Morning light exposure (Yes = 1, No = 0)	0.58	1.79 (0.74 ∼4.35)	0.20
Bedtime mobile phone use (Yes = 1, No = 0)	1.36	3.88 (1.60 ∼9.43)	0.003
Model: p=0.052			

Harm avoidance	B	Exp (B) (95% CI)	p
Gender (Female = 1, Male = 2)	0.26	1.29 (0.46 ∼3.63)	0.63
Age	-0.012	0.99 (0.96 ∼1.02)	0.48
Light preference (Brightness = 1, Darkness = 2)	1.36	3.91 (1.27 ∼12.10)	0.018
Morning light exposure (Yes = 1, No = 0)	-0.019	0.98 (0.40 ∼2.44)	0.97
Bedtime mobile phone use (Yes = 1, No = 0)	0.70	2.01 (0.87 ∼4.65)	0.11
Model: p=0.056			

Reward dependence	B	Exp (B) (95% CI)	p
Gender (Female = 1, Male = 2)	-0.64	0.53 (0.20 ∼1.42)	0.21
Age	-0.017	0.98 (0.95 ∼1.02)	0.31
Light preference (Brightness = 1, Darkness = 2)	-0.75	0.47 (0.18 ∼1.22)	0.13
Morning light exposure (Yes = 1, No = 0)	0.65	1.92 (0.82 ∼4.49)	0.13
Bedtime mobile phone use (Yes = 1, No = 0)	0.041	1.04 (0.46 ∼2.37)	0.93
Model: p=0.12			

Persistence	B	Exp (B) (95% CI)	p
Gender (Female = 1, Male = 2)	-0.18	0.84 (0.30 ∼2.34)	0.74
Age	-0.022	0.98 (0.95 ∼1.01)	0.21
Light preference (Brightness = 1, Darkness = 2)	-0.90	0.41 (0.15 ∼1.08)	0.072
Morning light exposure (Yes = 1, No = 0)	0.80	2.22 (0.94 ∼5.26)	0.069
Bedtime mobile phone use (Yes = 1, No = 0)	-0.77	0.46 (0.20 ∼1.10)	0.081
Model: p=0.036			

Self-directedness	B	Exp (B) (95% CI)	p
Gender (Female = 1, Male = 2)	-1.08	0.34 (0.12 ∼1.00)	0.05
Age	0.012	1.01 (0.98 ∼1.05)	0.51
Light preference (Brightness = 1, Darkness = 2)	-1.26	0.28 (0.10 ∼0.80)	0.017
Morning light exposure (Yes = 1, No = 0)	0.27	1.31 (0.52 ∼3.28)	0.58
Bedtime mobile phone use (Yes = 1, No = 0)	-0.95	0.39 (0.16 ∼0.92)	0.031
Model: p=0.006			

Cooperativeness	B	Exp (B) (95% CI)	p
Gender (Female = 1, Male = 2)	-1.53	0.22 (0.071 ∼0.66)	0.007
Age	0	1.00 (0.97 ∼1.04)	0.99
Light preference (Brightness = 1, Darkness = 2)	-0.73	0.48 (0.18 ∼1.28)	0.15
Morning light exposure (Yes = 1, No = 0)	0.55	1.73 (0.72 ∼4.20)	0.23
Bedtime mobile phone use (Yes = 1, No = 0)	-0.23	0.80 (0.35 ∼1.83)	0.60
Model: p=0.023			

Self-transcendence	B	Exp (B) (95% CI)	p
Gender (Female = 1, Male = 2)	0.033	1.03 (0.38 ∼2.81)	0.95
Age	0.015	1.02 (0.98 ∼1.05)	0.38
Light preference (Brightness = 1, Darkness = 2)	-0.22	0.81 (0.31 ∼2.07)	0.66
Morning light exposure (Yes = 1, No = 0)	0.47	1.60 (0.68 ∼3.78)	0.29
Bedtime mobile phone use (Yes = 1, No = 0)	-0.74	0.48 (0.21 ∼1.07)	0.072
Model: P=0.16			

Exp (B) = odds ratio.

## 4 Discussion

The main findings were that self-directedness was significantly associated with brightness preference and no bedtime mobile phone use. Although our hypotheses were that self-transcendence but not self-directedness may be associated with brightness preference and that harm avoidance may be associated with darkness preference, all hypotheses were not supported. Rather, self-directedness may be associated with brightness preference. On one hand, hyperthymic temperament is characterized by terms such as ‘cheerful,’ ‘overoptimistic,’ ‘overtalkative,’ ‘warm,’ ‘people-seeking,’ ‘extroverted,’ ‘overconfident,’ ‘self-assured,’ and ‘high energy level.’ (32, 33). This temperament has been reported to be associated with positive emotion (34), high flexibility to stress (35, 36), suppression of daily stress (37), and lowering of hopelessness (35, 36). In addition, several studies have reported that hyperthymic temperament prevents suicidal behavior and ideation (38, 39). On the one hand, self-directedness is characterized by terms such as ‘self-esteem,’ ‘responsible,’ ‘reliable,’ ‘goal-oriented,’ ‘constructive,’ ‘purposeful,’ ‘resourceful,’ ‘self-accepting,’ and ‘disciplined,’ with self as an autonomous individual (1, 40). This temperament has been reported to enhance emotion and satisfaction in life, social support, and mental health (41). In addition, self-transcendence is characterized by terms such as ‘spiritual,’ ‘unpretentious,’ ‘humble,’ and ‘fulfilled.’ Individuals with self-transcendence conceive themselves as integral parts of the universe as a whole, and this quality is adaptively advantageous when such people are confronted with suffering, illness, or death, which is inevitable with advancing age. Further, this trait has been reported to be associated with subjective well-being, resilience, and positive mental health (42). Taken together, both hyperthymic temperament and self-directedness have positive and realistic aspects, while self-transcendence has positive and transcended aspects. This is in line with the findings that harm avoidance scores were significantly and negatively associated with hyperthymic temperament scores and self-directedness scores, but not with self-transcendence scores after adjustment for relevant factors. Probably, these findings may be associated with the present findings that self-directedness but not self-transcendence was significantly associated with brightness preference.

A preference for darkness may lead to reduced light exposure, which may be associated with the onset and/or exacerbation of depression. Conversely, a preference for brightness may increase light exposure, potentially contributing to the prevention and/or improvement of depression. Therefore, individuals with high harm avoidance may be more susceptible to depression due to reduced light exposure from a preference for darkness, whereas those with high self-directedness may be less likely to experience or may recover from depression due to a greater light exposure from a preference for brightness. This aligns with previous findings that self-directedness may mitigate the effect of depressive mood on suicide risk (2) and that high harm avoidance and low self-directedness are predictors of depression and suicidal ideation (3). Furthermore, these results partially support prior research suggesting that non-remitters of depression tend to have high harm avoidance and low self-directedness, whereas non-responders exhibit high harm avoidance (4). Additionally, high resilience has been associated with low harm avoidance in depression and bipolar disorder and with high self-directedness in healthy individuals (5). Komasi et al. (6) suggested that high harm avoidance and low self-directedness may play a fundamental role in psychopathology in a meta-analysis, Oh and Cloninger (8) showed that patients with anxiety disorder + depressive disorder had harm avoidance was highest and persistence and self-directedness were lowest (i.e., they were lowest in Resilience). Erdem and Bahadir (10) showed that ADHD participants scored higher in novelty seeking and harm avoidance, while controls exhibited greater persistence and self-directedness. Overall, these suggest that high harm avoidance and low self-directedness may be associated with vulnerability of depression, anxiety disorder, and ADHD. Although the model of harm avoidance (**Table 3**) was not significant in the present study, the significance was nearly significant (p=0.056) in which model darkness preference was significant (p=0.018). These findings modestly suggest the possible association between harm avoidance and darkness preference.

Our recent study (25) showed that depressive, cyclothymic, and anxious temperaments were significantly associated with a preference for darkness over brightness, whereas hyperthymic temperament was significantly associated with a preference for brightness over darkness using Temperament Evaluation of Memphis, Pisa, Paris and San Diego-auto questionnaire version (TEMPS-A). Probably, there may be common aspects between self-directedness by TCI and hyperthymic temperament by TEMPS-A, which may be associated with brightness preference. Of interest, PRS for depression did not predict trajectories of novelty seeking (9). Therefore, there is a possibility that novelty seeking is not only congenital but also acquired. As aforementioned, it seems likely that light may maintain hyperthymic temperament in a dose-dependent manner and conversely individuals with hyperthymic temperament may prefer light, suggesting bidirectional relationship between hyperthymic temperament and light.

As for the association between self-directedness and no bedtime mobile phone use, the reason is uncertain. On one hand, hyperthymic temperament scores were not significantly associated with bed time mobile phone use (25). On the other hand, although the model of novelty seeking (**Table 3**) was not significant in the present study, the significance was nearly significant (p=0.052) in which model bedtime mobile phone use was significant (p=0.003). These findings modestly suggest a possible association between novelty seeking and bedtime mobile phone use. These findings may be due to the association between novelty seeking and seeking tendencies and the association between self-directedness and release from other viewpoints such as bedtime mobile phone.

As limitations, first, the number of subjects, particularly male subjects, was small. The sample is predominantly female (83%) and middle-aged, which limits generalizability. Second, we used the old version of TCI instead of TCI-R. Third, we used the very simple questions about light preference, morning light exposure, and bedtime mobile phone use, which could not have detailed information. Fourth, the adjusted R^2^ values were small in the multiple regression analyses, suggesting that the effects of light preference on temperament and character traits are modest.

## 5 Conclusion

The present findings suggest that self-directedness may be associated with brightness preference and no bedtime mobile phone use. Further studies are required to determine the causal relationships.

## Data Availability

The raw data supporting the conclusions of this article will be made available by the authors, without undue reservation.
